# Assessing Myocardial Architecture: The Challenges and Controversies

**DOI:** 10.3390/jcdd7040047

**Published:** 2020-10-29

**Authors:** Peter Agger, Robert S. Stephenson

**Affiliations:** 1 Comparative Medicine Lab, Department of Clinical Medicine, Aarhus University, 8220 Aarhus N, Denmark; 2 Department of Pediatrics, Randers Regional Hospital, Skovlyvej 15, 8930 Randers NE, Denmark; 3 Institute of Clinical Sciences, College of Medical and Dental Sciences, University of Birmingham, Birmingham B15 2TT, UK; r.s.stephenson@bham.ac.uk

**Keywords:** review, diffusion tensor imaging, micro computed tomography, heart, methodology, myocyte orientation, myocardial aggregation

## Abstract

In recent decades, investigators have strived to describe and quantify the orientation of the cardiac myocytes in an attempt to classify their arrangement in healthy and diseased hearts. There are, however, striking differences between the investigations from both a technical and methodological standpoint, thus limiting their comparability and impeding the drawing of appropriate physiological conclusions from the structural assessments. This review aims to elucidate these differences, and to propose guidance to establish methodological consensus in the field. The review outlines the theory behind myocyte orientation analysis, and importantly has identified pronounced differences in the definitions of otherwise widely accepted concepts of myocytic orientation. Based on the findings, recommendations are made for the future design of studies in the field of myocardial morphology. It is emphasised that projection of myocyte orientations, before quantification of their angulation, introduces considerable bias, and that angles should be assessed relative to the epicardial curvature. The transmural orientation of the cardiomyocytes should also not be neglected, as it is an important determinant of cardiac function. Finally, there is considerable disagreement in the literature as to how the orientation of myocardial aggregates should be assessed, but to do so in a mathematically meaningful way, the normal vector of the aggregate plane should be utilised.

## 1. Background

Myocardial architecture and cardiac function are closely linked [1,2,3,4,5,6,7]. Hence, the anatomy of the heart and the cellular construction of the myocardium has been the focus of research for centuries [8]. Traditionally, histology has been the method of choice [9,10,11], but owing to its two-dimensional nature, this technique fails to visualise the myocardial mass in its entirety. It has long been recognised that the myocardium is a highly complex three-dimensional syncytium [12,13], thus it is preferable to investigate its architecture using tools capable of representing this three-dimensionality. Such tools have been provided in the shape of diffusion tensor imaging [14,15], computed tomography [16,17,18], confocal microscopy [19] and ultrasound [20], with diffusion tensor imaging and computed tomography being the most prevalent and valid methods for quantifying myocardial architecture in three dimensions.

**Figure 1:**
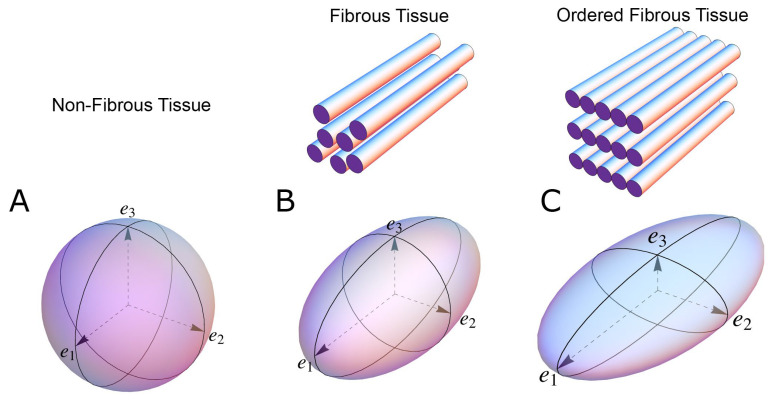
The shape of the diffusion tensor in different tissue environments. (**A**) Showing that all eigenvectors have equal magnitude in non-fibrous tissue resulting in a spherical shaped diffusion tensor. (**B**) Showing how, in fibrous tissue, the diffusion tensor takes on an ellipsoid shape when the magnitude of the primary eigenvector (e_1_) increases relative to the secondary eigenvector (e_2_) and tertiary eigenvector (e_3_). (**C**) In ordered tissue, the diffusion tensor can take on a flattened ellipsoid shape whereby the secondary eigenvector (e_2_) has a larger magnitude than the tertiary eigenvector (e_3_).

Since the beginning of the 1990s, diffusion tensor magnetic resonance imaging has been extensively used in the experimental setting to characterise myocardial architecture in both autopsied [21] and beating hearts [22]. Even though several independent groups have used this imaging technique for more than 20 years, there is still no consensus on the appearance of the myocardial microstructure, the way in which we quantify the orientation of the cells, nor on interpretations relative to physiology and pathology [7]. The main principle behind quantification of myocardial architecture is the measurement of cardiomyocyte orientation. The cardiomyocytes are elongated cells measuring approximately 100 by 20 by 20 microns, and the overall goal is to assess the orientation of their long axis, as this is the main direction of force transmission. Diffusion tensor imaging achieves this by quantifying the direction and magnitude of Brownian motion of water molecules, that is the spontaneous diffusion occurring in both viable and fixed tissues [15]. In short, the result is presented as a three-dimensional mathematical construct called a tensor, the dimensions of which reflect the likely pattern of diffusion, itself a validated surrogate of the myocyte orientation (Figure 1). Likewise, computed tomography describes the myocardial morphology by the use of a tensor, but in this case the tensor is calculated by variations in x-ray attenuation within the tissue, where the direction of least difference is deemed to represent the longitudinal course of the myocyte chains. Consequently, this is referred to as a “structure tensor” rather than a “diffusion tensor” [18,23]. 

In general, a tensor is described using its three orthogonal axes. These are called eigenvectors, which are designated as being primary (e_1_), secondary (e_2_), and tertiary (e_3_). A complete mathematical description of a tensor is beyond the scope of this paper, but the interested reader is advised to consult specific literature dedicated to this matter [24]. To avoid confusion, it is important to note that owing to the underlying mathematical principles of tensor calculation, the long axis of the cardiomyocytes corresponds with the primary eigenvector in the diffusion tensor. Whilst in the structure tensor, the tertiary eigenvector aligns with the cardiomyocytes’ long axes [25,26]. The subsequent mathematical determination of myocyte orientation is identical for the two techniques. It has been rightfully argued that the main drawback of diffusion tensor imaging is its inability to assess the anatomy directly, instead using the spontaneous diffusion of water as a surrogate measure of the myocyte orientation [27]. Conversely, computed tomography, together with high-resolution conventional magnetic resonance imaging, provides the opportunity to evaluate the myocardial architecture based on tracking of actual anatomical features or “structures” [17,18]. This is an obvious advantage of computed tomography, but diffusion tensor imaging also holds important advantages. First of all, it is the only technique that currently holds potential as a clinical tool [28], and secondly it is the only validated and widely used methodology for assessing the orientation of the myocardial aggregates [7,17,29,30]. 

To understand why this is the case, we need to dig a little deeper into the concepts of tissue diffusion. In an environment without cell membranes and other diffusion boundaries, the water molecules are equally likely to diffuse in all directions, thus the diffusion tensor assumes the shape of a sphere (Figure 1A). In biological tissues, whether within a cell or in the surrounding extracellular matrix, diffusion will be hindered mainly by the hydrophobic cell membranes. In tissues consisting of non-isotropic cells, such as in the brain or in muscles, the water diffuses most easily along the long axis of the cells. If the cells are grouped in common directional alignment, the tensor becomes an ellipsoid, with its long axis in the same direction as the common cellular long axis (Figure 1B). This configuration is typified by skeletal muscle, and by the long axonal tracts of the nervous system, particularly the spinal cord [31]. If the cells are also grouped into secondary substructures of reasonably regular shape, the signal from the extracellular water might cause differences in the magnitude of the secondary and tertiary eigenvectors. This is particularly the case when the cells are arranged so as to compartmentalise themselves in laminar fashion. As the myocytes in the laminar structure are aggregated tightly together, the water molecules are more likely to diffuse across this structure than through it. Thus, the secondary eigenvector will align with the plane of the laminar substructure, as this is the direction of greatest diffusion magnitude orthogonal to the primary eigenvector. Consequently, the diffusion tensor will assume a more flattened ellipsoid shape (Figure 1C). It is now well established that, in the myocardium, the primary eigenvector of the diffusion tensor follows the orientation of the chains of cardiomyocytes [21,32,33,34,35]. It has then been suggested that the secondary eigenvector follows the surface of the flattened groupings of cardiomyocytes, often described as myocardial sheets [32], laminae [9], sheetlets [36], lamellae [37], lamellar units [7,38] or aggregated units of cardiomyocytes [30]. This disagreement in nomenclature can be attributed to the current lack of a suitable three-dimensional anatomical description of these sub-structures. It is inherently difficult to assign a suitable name to a structure whose anatomical extent is unknown. Given our knowledge of their structural heterogeneity in size and thickness, we believe “myocardial aggregates”, as a name, currently provides the most suitable denomination. It was LeGrice and co-workers [39] who originally posited the existence of myocardial aggregates using electron microscopy. Computed tomography [8], confocal microscopy [19], ultrasound [40], and even photographically based methods [41], have also been used to evaluate the micro-anatomical features of the myocardial aggregates. None of these methods, however, can assess the aggregate normal vector, which we believe is key to calculating the precise orientation of the myocardial aggregates. To date, the normal of the myocardial aggregations has been assessed using diffusion tensor imaging [4], structure tensor calculation [42] and conventional histology [11]. Despite this, the most prominent approach is to assess myocardial aggregate orientation using the in-plane secondary eigenvector, which we claim is not founded in mathematical theory.

Many investigators are now exploring the remodelling of myocyte orientation in disease, with results now emerging characterising changes in hypertrophic and dilatated cardiomyopathies, and congenital malformations [16,26,43,44,45]. This has led to the desire to explore the prognostic and diagnostic potential of myocyte orientation analysis [28], thus knowledge of its technical limitations and methodological inconsistencies is paramount. This review will discuss the variability and validity of current practices in the field and propose guidance to establish methodological consensus in the field. 

## 2. Assessing Myocardial Architecture

### 2.1. Establishing Reference Points

**Figure 2:**
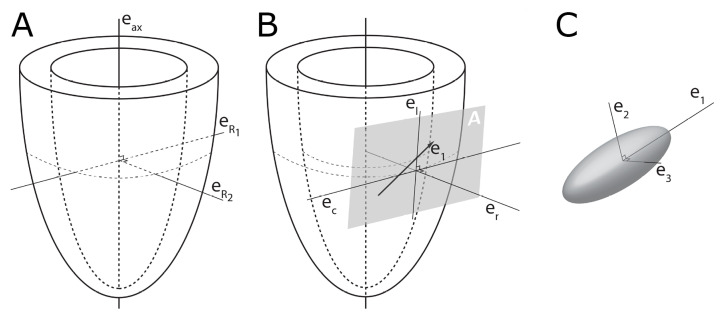
Establishment of the reference planes. (**A**) Illustrates the global geometric coordinate system defining the position of the left ventricle. It is defined by the orthonormal basis [$\vec{e_{ax}},\vec{e_{R_{1}}},\vec{e_{R_{2}}}$] where $\vec{e_{ax}}$ is the left ventricular long axis and $\vec{e_{R_{1}}}$ and $\vec{e_{R_{2}}}$ being orthogonal radial vectors, which are not used in the determination of myocyte orientations. (**B**) Subsequently, the local wall coordinate system is defined based on the orthonormal basis [$\vec{e_{r}},\vec{e_{c}},\vec{e_{l}}$], where $\vec{e_{r}}$ is the local radial vector, i.e., the normal of the epicardial tangential plane A, $\vec{e_{l}}$ is the local longitudinal vector, i.e., the normal of the local horizontal plane, and lastly $\vec{e_{c}}$ is the local circumferential vector. (**C**) Finally, the diffusion tensor is defined by a sub-local coordinate system based on the orthonormal bases [$\vec{e_{1}},\vec{e_{2}},\vec{e_{3}}$], which are the three eigenvectors. Modified from [25].

**Figure 3:**
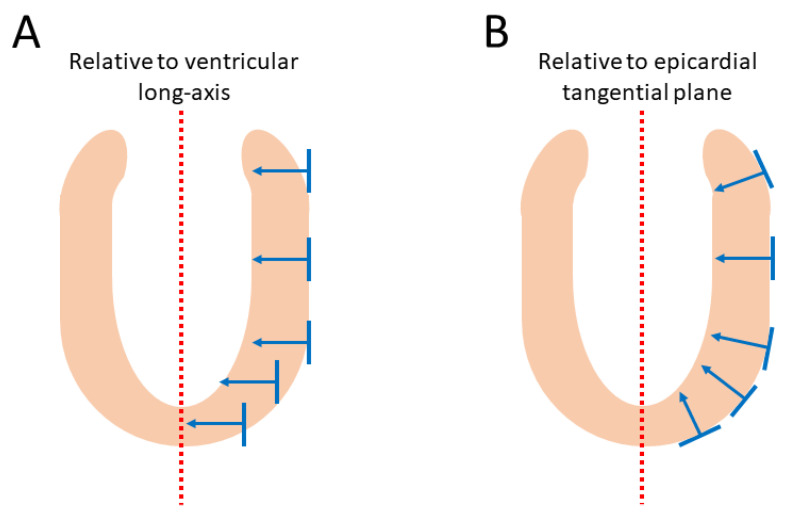
Epicardial wall normalisation. Schematic illustration of the influence of epicardial curvature on the quantification of myocyte orientation. The figure shows how myocyte orientation can be assessed either relative of the left ventricular long axis (**A**) or the epicardial tangential plane (**B**). Owing to the rounded shape of the ventricular cavities, the myocardial contractile forces work perpendicular to the epicardial surface. Therefore, to measure myocyte orientation accurately throughout the entire myocardium, we should quantify relative to the epicardial curvature (**B**). If we assess myocytes orientation relative to the left ventricular long axis (**A**), we do not compensate for the epicardial curvature, thus myocyte orientation will not correlate with wall deformation.

**Figure 4:**
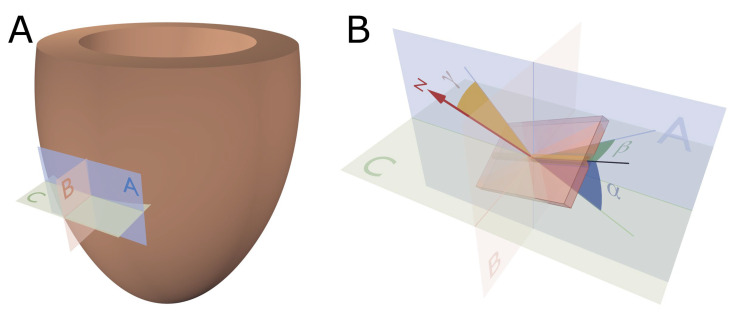
Reference planes and angle definitions. (**A**) showing a schematic of the left ventricle with the local orthogonal reference planes aligned with the epicardium. Plane A is parallel to the epicardial tangential plane, while the orthogonal plane B is parallel to the left ventricular long axis. Consequently, plane C is orthogonal to both planes A and B and is often referred to as the local “horizontal” plane. (**B**) Outlines our recommended angle definitions. The helical angle α is the angle between the primary eigenvector (black line) and plane C. The intrusion angle β is the angle between the primary eigenvector and plane A. Lastly, the aggregate angle is measured using the aggregate plane normal (N) assessed against the epicardial tangential plane A. The unit of aggregated cardiomyocytes is depicted as the yellow box, which is a schematic oversimplification.

In order to describe the orientation of the chains of cardiomyocytes, it is agreed that unique points of reference are needed. There is general agreement in the published literature that, in the first instance, the global orientation of the heart itself should be described using the left ventricular long axis. This is usually achieved by placing a line between the apex and the fibrous continuity between the leaflets of the aortic and mitral valves [39,45,46,47]. An alternative approach is to interpolate a line between the centres of the ventricular cavity in a series of short axis images [25,48,49,50,51]. The left ventricular long axis, along with two orthogonal radial vectors (e_R1_ and e_R2_), then provides the global geometric coordinate system for defining the position of the heart (Figure 2A). In some studies, these are the only points of reference used when assessing myocytic orientation [51,52,53]. Systolic mural thickening, however, which is the main rearrangement of the myocardium through the cardiac cycle, predominantly occurs relative to a radial axis at right angles to the epicardium [54]. Hence, it makes sense physiologically that angles be assessed relative to the epicardial tangential plane (Figure 3), as this provides the most relevant information concerning cardiodynamics [17,25]. Therefore, once the orientation of the left ventricle is established using the left ventricular long axis (e_ax_), a second local coordinate system should be introduced, which can be positioned relative to the individual region of interest in the heart (Figure 2B). On this basis, one can propose three orthogonal vectors, which can be considered as being longitudinal (e_l_), circumferential (e_c_), and radial (e_r_). The circumferential vector is orthogonal to the left ventricular long axis, and tangential to the epicardium. The longitudinal vector is orthogonal to the circumferential vector, and again tangential to the epicardium. The radial vector is orthogonal to both the longitudinal and circumferential vectors. It is also normal to the epicardium, or more accurately normal to the epicardial tangential plane [55] (Figure 2B). For the sake of ease of analyses, these three vectors of the local coordinate system are translated into three planes of reference (Figure 4). In Figure 4 the epicardial tangential plane (A) is defined by the longitudinal and the circumferential vectors (e_l_ and e_c_), while the circumferential and radial vectors (e_c_ and e_r_) define the local horizontal plane (C). The radial and the longitudinal vectors (e_r_ and e_l_) define the local sagittal plane (B). 

It serves to mention that using only the left ventricular long axis as a reference point, and thus presuming that the left ventricle can be contemplated as cylindrical in shape for analytical purposes, will render skewed results when investigating the myocardial architecture at the base and apex of the ventricular cone. In these areas, the epicardium is in reality far from parallel with the long axis. Ideally, therefore, a subset of reference points is needed for each region of interest as described above. These reference vector definitions have already been used by different groups [27,34,56,57], but are far from universally employed. This so-called epicardial normalisation must be adopted to account for the inherent epicardial curvature, and to provide the most physiologically meaningful estimate of myocyte and aggregate orientation (Figure 3) [25].

### 2.2. The Helical Angle

By using “standard” planes of reference based on “unique” reference points (Figure 2 and Figure 4), it becomes possible to quantify myocyte orientation by analysing the eigenvector corresponding to the myocyte chain’s long axis. In order to completely describe the orientation of a vector in a three-dimensional space, one needs to assess its orientation relative to two of the three reference planes discussed above [58]. It is Streeter and his colleagues who are usually credited with introducing the notion of the helical angle, which assesses myocyte orientation relative to the equatorial/horizontal plane of the ventricular cone [59,60]. However, this notion of change in myocyte angle relative to transmural position goes further back in time [61]. The idea that such angulation could be assessed relative to the horizontal plane was originally introduced by Feneis [62]. The notion was later endorsed by Hort [63] when the latter performed his extensive investigations of myocardial structure. Such helical angles are considered positive in the sub-endocardium, approximately zero in the mid-wall, and negative in the sub-epicardium. 

**Figure 5:**
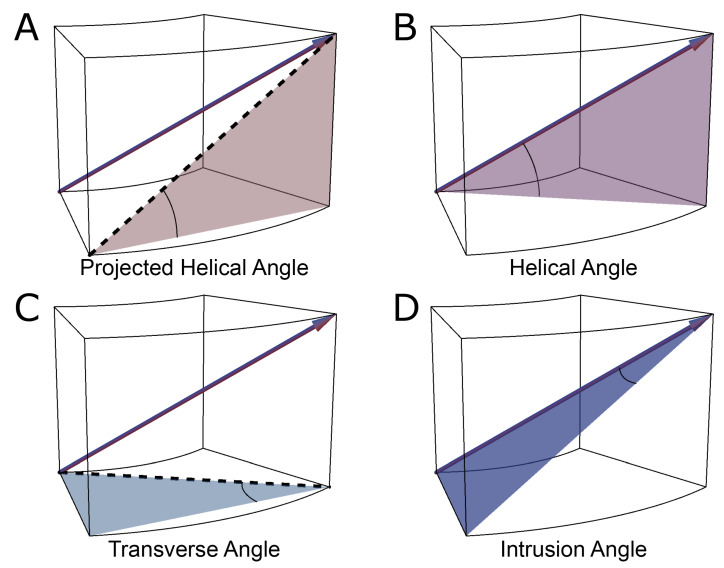
Commonly used angles in analysis of myocardial architecture. This figure illustrates the most commonly used definitions of helical and ‘transmural’ angulations with projection (**A**,**C**) and without projection (**B**,**D**). In this schematic illustration, the principal orientation of the cardiomyocytes, that is the primary eigenvector (arrow in all images), is depicted within a block of myocardium with the epicardium facing out of the page. (**A**) The projected helical angle is defined as the angle between the local horizontal plane and the projection of the primary eigenvector onto the epicardial tangential plane. (**B**) The helical angle is the angle between the primary eigenvector and the local horizontal plane. (**C**) The transverse angle is defined as the angle between the epicardial tangential plane and the projection of the primary eigenvector onto the local horizontal plane. (**D**) The intrusion angle is defined as the angle between the primary eigenvector and the epicardial tangential plane.

The assessments by Streeter and his colleagues, along with those performed by his predecessors, were conducted manually, either by dissection, histology or both. Such methods were then used by others examining human [64] and animal specimens [65]. In 1992, Bovendeerd and colleagues investigated the mechanics of myocardial architecture with a Finite Element model [66]. They sought to replicate the assessments of Streeter by projecting the paths of the myocyte chains onto the epicardial tangential plane before measuring the helical angle (Figure 5). Apart from a few notable exceptions [34,52,67,68], this “projection method” has become the standard approach for assessing helical angles [28,69]. Of the alternative strategies, the one used by Geerts and co-workers is of interest, since they assessed the helical angle as the angle between the primary eigenvector and the local horizontal short axis plane, and thus avoided the need for projection [52] (Figure 5). 

It has long been suspected that the ventricular cardiomyocytes change their orientation through the cardiac cycle to accommodate the dynamic wall deformation required to eject blood. Streeter and colleagues investigated this in dogs, they found no changes in the helical orientation of the myocytes in systole versus diastole [60]. While Streeter’s assessments were based on histologic evaluation, Dou and co-workers investigated the same phenomenon more than three decades later in humans using diffusion tensor imaging [70]. Contrary to the findings of Streeter and colleagues, they found the helical orientation to change through the cardiac cycle, going from a more circumferential orientation in diastole towards a more longitudinal orientation in systole. This finding has subsequently been confirmed in humans [36] and in rats [48,50], all using diffusion tensor imaging. In a recent and more detailed study of the entire heart by Omann and colleagues, the helical angle was found to only change significantly in the left ventricle and the septum, whereas no significant change was found in the right ventricle [4]. In the midwall and endocardial third of the left ventricle, the helical angle increased 15 to 20 degrees during contraction. This is comparable to the results of Chen and associates. According to their analyses the helical angle changes from approximately ±50 degrees in diastole to ±65 degrees in systole [48]. Comparing these data to those obtained in humans shows good agreement in some studies [67,71,72], while others find completely different extremes [73,74]. These inconsistencies are likely caused by differences in resolution, differences in the definition of the angle itself, and the use of projected angle calculations. This is a pertinent issue in the field. Owing to a lack of consensus regarding angle definitions and the associated quantification, many research teams would produce varying results even when analysing the same heart.

### 2.3. Transmural Orientation

Irrespective of the precise angle definition, once the helical orientation of the cardiomyocytes is determined relative to the local horizontal plane of the left ventricle, it also becomes necessary to consider any change in transmural orientation relative to the epicardial tangential plane (Figure 5). Once these two angles are defined, one has established the precise orientation of the cardiomyocytes. Thus, it is the combination of the helical and transmural orientations that provides the complete anatomical description of the orientation of the cardiomyocyte chains [25]. The notion of transmural angulation (i.e., across the wall) was also investigated by Streeter and his colleagues, and consequently named by them: the angle of imbrication [75]. This term, however, has not survived the passage of time. A significant number of morphological studies, nonetheless, have explicitly denied the existence of populations of cardiomyocytes aggregated together with transmural orientation [10,34,36,51,66,67,68,76,77,78]. Evidence now confirms that transmural angulations do indeed exist [4,13,55,79], and it is suggested they play a key role in cardiac function [54,56,80]. The notion of mural antagonism and its functional significance [2,80] is yet to gain field-wide acclaim; this may be due, in part, to the historical dogma attached to the existence of transmurally arranged cardiomyocytes. 

In various investigations, transmural angulation of the cardiomyocytes chains has been assessed either as the angle of intrusion, or the transverse angle (Figure 5). These two angles differ one from the other, with the transverse angle projected onto the local horizontal plane before it is then assessed relative to the epicardial tangential plane, while the angle of intrusion is measured without projection. The angle of intrusion has been used sparingly [55,56,79,81], while the transverse angle has been more commonly assessed [32,33,48,50,52,53,71,73,82,83]. We have previously discussed the limitations inherent to projection-based quantification [25]. Although it is argued that projected angles may be informative in a functional context, for example, when compared with measurements of strain [56,84], they actually act to mask the true anatomical arrangement of the myocyte chains. The concept of projection error is discussed further in Section 2.5.

### 2.4. Myocardial Aggregate Orientation 

**Figure 6:**
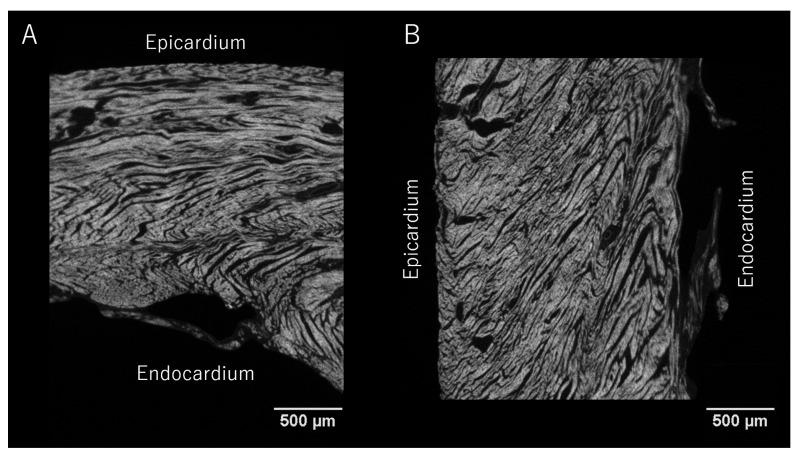
Micro-computed tomography of the myocardium. Contrast enhanced micro-computed tomography images of a sample preparation taken from the posterior-basal region of a rabbit left ventricle showcasing the aggregations of cardiomyocytes. Panel (**A**) shows the sample in short axis view, panel (**B**) shows the corresponding four-chamber view. Scale bars represent 500 µm. The isotropic spatial resolution is approximately 4 µm.

Anatomists have long discussed the existence of anatomical subgroupings or aggregations of cardiomyocytes within the myocardium [12,85,86]. The extent of such myocardial aggregations and their role in both the compartmentalisation and deformation of the ventricular walls remain two of the most significant controversies in cardiac morphology. These aggregations are of major importance in the continuous rearrangement of the myocardium through the cardiac cycle [4,28]. Studies of the supporting fibrous matrix have shown that the cardiomyocytes are packed together in functional subunits [87]. This notion of packing led LeGrice and colleagues to propose that the ventricular walls are organised in anatomical subunits of a laminar nature [9]. They stated that the individual myocardial aggregates were arranged in relatively uniform fashion, with a thickness of four to six myocytes. This notion was then endorsed by Scollan and colleagues [32]. As can be seen from Figure 6, the arrangement of the myocardial aggregates is more of a complex heterogenous mesh of interconnected aggregations. The sub-organisation of the myocardium into myocardial aggregates, in our opinion, is an indisputable fact. However, the exact anatomical dimensions of the aggregations, and their alignment within the ventricular walls, are less easily elucidated. Although this conundrum remains unresolved, it has been speculated that the units are joined together in an infinite heterogeneous branching continuum, with no discernible beginning or end [8,17,30,37,88]. It is hypothesised that this so-called cardiac mesh allows the aggregations of myocytes to slide one against the other during systolic mural thickening [6,10,39,65,89]. Evidence also suggests that the organisation into structural subunits determines the properties of electric conduction through the myocardium [90,91]. Even though the precise micro-anatomical organisation of such aggregations of cardiomyocytes is far from clarified, much emphasis has been placed on their three-dimensional orientation. 

**Figure 7:**
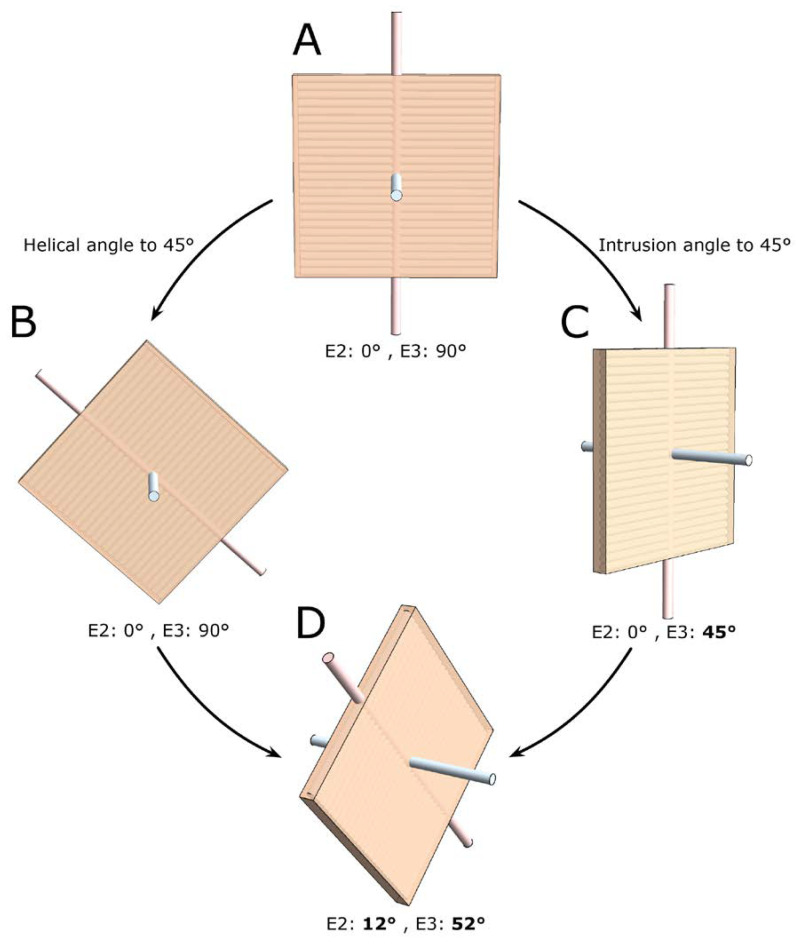
Differences in the quantification of myocardial aggregate orientation. In the literature, myocardial aggregate orientation is assessed using either the eigenvector situated within the aggregate plane, in diffusion tensor imaging referred to as the secondary eigenvector (E2) i.e., the pink shaded rod, or it is assessed using the aggregate plane normal, in diffusion tensor imaging referred to as the tertiary eigenvector (E3) i.e., the light blue shaded rod. This schematic shows a myocardial aggregate (beige box) made up of cardiomyocyte chains (depicted as lines running across the box). In panel (**A**) the aggregate is orientated parallel to the myocardial surface, with the helical and intrusion angle at 0 degrees. When adopting the most widely used E2-angle definition [36] this configuration results in an E2 angle of 0 degrees, conversely when using the E3-angle definition [25] the E3 angle is 90 degrees. Assigning a helical angle of 45 degrees to the cardiomyocyte chains, as shown in panel (**B**), changes neither the E2 nor the E3 angle. However, when we assign an intrusion angle to the myocyte chains, as shown in panel (**C**), the aggregate now angles towards the endocardium as is the case during myocardial thickening. This crucial reorientation is detected by the E3 angle, which changes to 45 degrees, the change is not detected by the E2 angle, which remains 0 degrees. If we assign both a 45 degree helical and a 45 degree intrusion angle to the cardiomyocyte chains, as shown in panel (**D**), despite this marked reorientation the E2 angle increases by only 12 degrees whereas the associated E3 angle is 52 degrees. This figure illustrates why the E3 angle more accurately measures aggregate transmurality and reorientation during wall thickening, and emphasises why the two cannot be readily compared.

Before function can be attributed in a meaningful fashion to the myocardial aggregates, it is necessary to appreciate both their extent and their orientation. The so-called “sheet angle” was introduced allegedly to provide such information. Early studies had used histological techniques in an attempt to characterise the orientation of the myocardial aggregates [9,10,11,77,92,93], even though their extent had not been established in three dimensions. It was in 1998 that Scollan and his associates pointed towards the non-random orientation of the secondary and tertiary eigenvectors in diffusion tensor imaging and hypothesised that this feature may be linked to the orientation of the myocardial aggregates [32]. There is absolutely no consensus in the literature regarding the definition of this angle [25]. Several investigators have again made use of projection when calculating the so-called sheet angles [32,36,50,57,70,81,94,95]; this introduces a “projection error” as described in Section 2.5. Only the works of Chen and colleagues [48], Kung and associates [34], and the work from our own group [4,25,43,44] have assessed the orientation of the myocardial aggregates without the use of projections. As when assessing all other types of myocytic angulations, eigenvector projection serves no relevant purpose in assessment of the orientation of the aggregates [25]. It is the tertiary eigenvector, being the normal of the aggregate plane (Figure 7), which should be used when adopting the diffusion tensor approach [25]. Conversely, when using the structure tensor approach, it is the primary eigenvector, being the normal of the aggregate plane, which should be adopted [96]. Myocardial aggregates are of a planar nature. The only mathematically correct way of describing the orientation of a plane in space is to use its normal vector (Figure 7). We encourage researchers in the field of myocardial morphology to adopt this mathematical logic. In further support of this ideology, it is a generally accepted notion that changes in the orientation of the myocardial aggregates aid in radial thickening of the ventricular walls [39]. It is therefore reasonable to conclude that the orientation of the aggregations should be assessed relative to the local epicardial tangential plane (Figure 3 and Figure 4). Thus, the most intuitive way of assessing the orientation of a myocardial aggregate, is to assess the angle between the normal vector of the aggregate and the epicardial tangential plane itself. This angle is depicted in Figure 4 as γ.

### 2.5. Consequences of Projected Angles 

**Figure 8:**
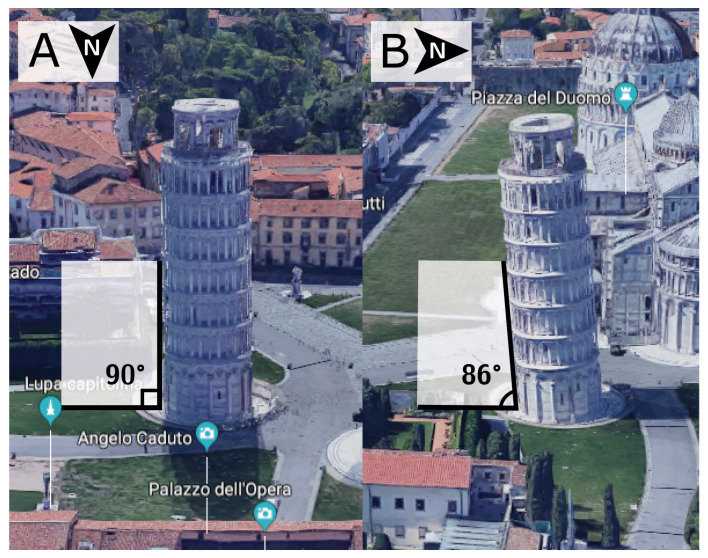
Projection artefact. The leaning tower of Pisa taken as an everyday example of projection artefact. The tower leans in a southward direction. Thus, when viewed from the north, the tower appears to be standing straight (**A**). When viewed from the east, however, the tower is obviously leaning (**B**). The straight appearance of the tower in panel A is an artefact brought upon by the projection of the tower into the camera lens. It would be inappropriate to use two-dimensional photography in an attempt to quantify the inclination of the tower. All projected angles in the setting of myocardial morphology are subject to projection artefact. ©2018 Google, Data SIO, NOAA, U.S. Navy, NGA, GEBCO, Landsat/Copernicus.

As we have already discussed, it is frequent to find studies where vectors have been projected onto reference planes prior to assessing their angulations [25]. If we now accept the fact that not all cardiomyocytes are arranged in surface parallel fashion, or in other words that myocyte chains exhibit a transmural orientation, we must also accept that projected angles are prone to bias or anatomically inaccurate results. Previously we assessed the consequences of projection when calculating helical, intrusion, and aggregate angle [25]. We showed the larger the intrusion angulation, the more the corresponding projected helical angle deviates away from its true value. The transmural orientation of cardiomyocytes in particular is very sensitive to projection, with the larger the helical angle, the greater the deviation of the projected transverse angle away from the non-projected intrusion angle. It was to circumvent such issues that Lunkenheimer and co-workers elegantly used circular knives to remove blocks of ventricular wall, thereby removing the influence of the helical angle when calculating the intrusion angle [13]. The artefact attributed to projection, is undisputable and a phenomenon we can easily recreate with everyday objects (Figure 8). It is surprising, therefore, that projection error has received so little attention in the existing literature [42]. 

The leaning tower of Pisa taken as an everyday example of projection artefact. The tower leans in a southward direction. Thus, when viewed from the north, the tower appears to be standing straight (A). When viewed from the east, however, the tower is obviously leaning (B). The straight appearance of the tower in panel A is an artefact brought upon by the projection of the tower into the camera lens. It would be inappropriate to use two-dimensional photography in an attempt to quantify the inclination of the tower. All projected angles in the setting of myocardial morphology are subject to projection artefact. ©2018 Google, Data SIO, NOAA, U.S. Navy, NGA, GEBCO, Landsat/Copernicus.

## 3. Conclusions

This review points towards several controversies in the field of myocardial architecture. Data obtained by high-resolution imaging can be analysed, displayed, and mathematically modified in myriads of ways. We can learn much that is novel from studies of myocardial micro-architecture, but currently there is little consensus in the literature as to how myocyte orientation is defined and quantified. For as long as disagreements prevail, we will remain unable to compare studies and draw valid anatomical, physiological, and clinical conclusions. Based on this review we suggest the following points be considered in future studies of myocardial architecture. Firstly, as myocardial thickening occurs perpendicular to the epicardium, its curvature should be taken into account when assessing myocyte orientations. Second, there is no need to project myocyte orientations prior to quantification. In fact, projection introduces considerable bias. Third, the transmural component of myocyte orientation should be assessed, as it is a major determinant of cardiac function. Fourth and last, the normal vector of the myocardial aggregates should be used when quantifying aggregate orientation.
